# High-quality *Lindera megaphylla* genome analysis provides insights into genome evolution and allows for the exploration of genes involved in terpenoid biosynthesis

**DOI:** 10.1093/hr/uhaf116

**Published:** 2025-04-29

**Authors:** Hongli Liu, Jing Liu, Yun Bai, Xinran Zhang, Qingzheng Jiao, Peng Chen, Ruimin Li, Yan Li, Wenbin Xu, Yanhong Fu, Jiuxing Lu, Xiaoming Song, Yonghua Li

**Affiliations:** College of Landscape Architecture and Art, Henan Agricultural University, Zhengzhou 450002, Henan, China; College of Landscape Architecture and Art, Henan Agricultural University, Zhengzhou 450002, Henan, China; School of Life Sciences/School of Basic Medical Sciences, North China University of Science and Technology, Tangshan 063210, Hebei, China; College of Landscape Architecture and Art, Henan Agricultural University, Zhengzhou 450002, Henan, China; College of Landscape Architecture and Art, Henan Agricultural University, Zhengzhou 450002, Henan, China; College of Landscape Architecture and Art, Henan Agricultural University, Zhengzhou 450002, Henan, China; Gannan Normal University, College of Life Sciences, Ganzhou 341004, Jiangxi, China; College of Landscape Architecture and Art, Henan Agricultural University, Zhengzhou 450002, Henan, China; Wuhan Botanical Garden, Chinese Academy of Sciences, Wuhan 430074, Hubei, China; School of Life Sciences/School of Basic Medical Sciences, North China University of Science and Technology, Tangshan 063210, Hebei, China; College of Landscape Architecture and Art, Henan Agricultural University, Zhengzhou 450002, Henan, China; School of Life Sciences/School of Basic Medical Sciences, North China University of Science and Technology, Tangshan 063210, Hebei, China; College of Landscape Architecture and Art, Henan Agricultural University, Zhengzhou 450002, Henan, China

## Abstract

*Lindera megaphylla*, a Lauraceae species, is valued for timber, horticulture, landscape architecture, and traditional medicine. Here, a high-quality genome of *L. megaphylla* was obtained at the chromosome level. A total of 96.77% of genomic sequences were mapped onto 12 chromosomes, with a total length of 1309.2 megabase (Mb) and an N50 scaffold of 107.75 Mb. Approximately, 75.91% of genome consists of repetitive sequences and 7004 ncRNAs were predicted. We identified 29 482 genes, and 28 657 genes were annotated. Gene family analysis showed expanded gene families were mainly involved in energy metabolism and cellular growth, while contracted ones were associated with carbohydrate metabolism and signal transduction. Our analysis revealed that *L. megaphylla* has undergone two rounds of whole-genome duplication (WGD). Our results revealed that volatile compounds in *L. megaphylla* leaves inhibited the growth of several fungi and bacteria. Fifty-two terpene synthase (TPS) genes were identified and classified into six subfamilies, with significant expansion observed in the TPS-b, TPS-f, and TPS-g subfamilies in *L. megaphylla*. Transcriptomic and metabolomic co-analysis revealed that 43 DEGs were correlated with 117 terpenoids. Further analysis revealed that *LmTPS1* was significantly correlated with caryophyllene oxide content. The overexpression of *LmTPS1* in transgenic tomato lines significantly increased the contents of β-caryophyllene and humulene, which further improved the resistance of transgenic tomato plants to common fungal and bacterial diseases. The integrated analysis of genome, metabolome, and transcriptome provides comprehensive insights into the evolution of *L. megaphylla* and clarifies the molecular mechanisms underlying the protective effects of caryophyllene against biotic stress.

## Introduction

Land plants are subjected to various adverse environmental conditions, such as pest and pathogen invasion, which cause damage to plant growth and decrease crop quality and yield [1, 2]. To overcome these complex biotic stresses, plants have generated strategies and defense mechanisms during their long evolution, operating at the morphological, physiological, biochemical, and molecular levels [1, 3]. Among these mechanisms, secondary metabolites produced by plants, including tannins, alkaloids, phenolics, steroids, flavonoids, and terpenoids, play essential roles due to their antioxidant and antimicrobial properties, helping plants cope with adverse environments [4].

**Figure 1:**
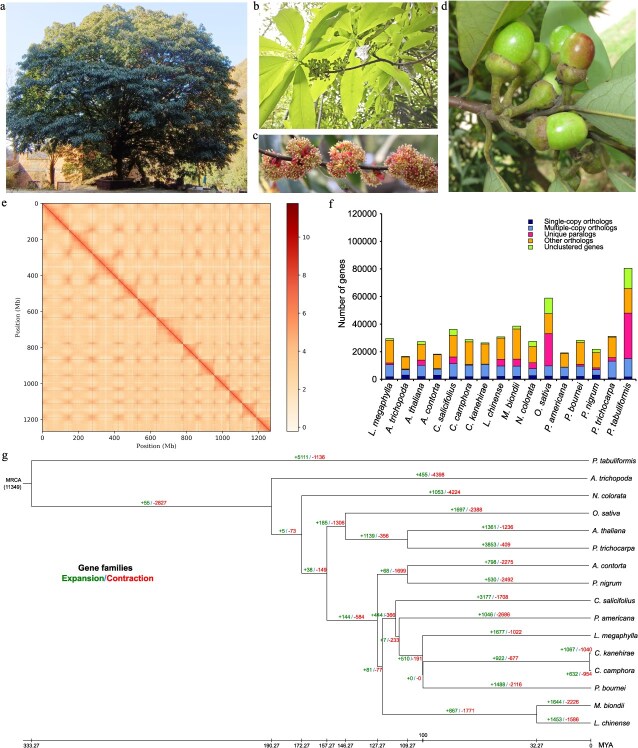
Phenotype, Hi-C contact map, and gene family analysis of the *L. megaphylla* genome. (a-d) Phenotypes of the whole plant, leaves, flowers, and fruits of *L. megaphylla*. (e) All-by-all interactions across the 12 chromosomes obtained by Hi-C sequencing within the *L. megaphylla* genome. (f) The number of genes in each gene family in *L. megaphylla* and 15 representative species. (g) Gene family expansion and contraction in *L. megaphylla* and 15 representative species.

Terpenoids, as important secondary metabolites, contribute to improving plant growth and responding to adverse stresses [5]. Two pathways, the methylerythritol phosphate (MEP) and mevalonate (MVA) pathways, are involved in regulating terpenoid biosynthesis in plants and are used to produce isopentenyl diphosphate and dimethylallyl diphosphate (IPP and DMAPP, respectively) [6]. Numerous studies have revealed that IPP and DMAPP are used as precursors of geranyl diphosphate, farnesyl diphosphate, and geranylgeranyl diphosphate (GDP, FDP, and GGDP, respectively), which are catalyzed by TPSs to form monoterpenes, sesquiterpenes, and diterpenes, respectively, under normal growth conditions [6, 7]. Modifications, including methylation, acylation, oxidation, and peroxidation, of TPS products improve physical properties and biological activities (e.g. antioxidant, anticancer, and antimicrobial activities), which play several ecological roles in attracting pollinators and providing chemical protection against microbes and plant-feeding insects [8]. In addition, TPS products and their derivatives are commonly used as pharmaceuticals (limonene, carveol, perillyl alcohol, and pyrethrins), flavorings (e.g. limonene, menthol, and santalene), fragrances, and high-quality liquid fuel alternatives (e.g. farnesene) [9]. Caryophyllene, an important terpenoid compound, contributes to modulating plant tolerance against diseases and pests and is used for treating anxiety and depression due to its anesthetic and anti-inflammatory properties [10, 11]. Previous studies revealed that *TPS* genes could participate in the biosynthesis of caryophyllene in many plants [12–14]. However, the systematic regulatory mechanisms underlying the role of caryophyllene in plant–pathogen interactions are still poorly understood.

The Lauraceae family, which includes 67 genera with more than 2500 species, contains many economically important species [15, 16]. To date, extensive comparative genomic studies have been performed on several Lauraceae species, including *Phoebe bournei* [17], *Litsea cubeba* [18], *Persea americana* [16], and *Cinnamomum kanehirae* [19]. *Lindera megaphylla,* a valuable traditional Chinese medicinal plant with 24 chromosomes (2*n* = 2*x* = 24) belonging to the *Lindera* genus of the Lauraceae family, is commonly distributed in subtropical/warm-temperate regions [20, 21]. Secondary metabolites produced by Lauraceae species, such as terpenoids, flavonoids, and alkaloids, have been used in the production of pesticides, industrial feedstocks, and antimicrobial drugs [15, 20, 21]. As research on the bioactive compounds in *L. megaphylla* has increased, researchers have focused mainly on the extraction, characterization, and exploration of potential applications of medicinal components such as D-dicentrine, (+)-dicentrine, and northalifoline [20, 22].

Transcriptome datasets encompassing various tissues or different developmental stages of *L. megaphylla* have been acquired, enabling in-depth functional genetic analyses of this species [23, 24]. In 2023, a genome sequence of *L. megaphylla* was acquired with a contig N50 length of 2.61 Mb, significantly advancing research on the breeding progress of *L. megaphylla* and comparative genomic analysis within the Lauraceae family [15]. A high-quality genome of *L. megaphylla* has gained significant importance for obtaining a more comprehensive gene set. Here, we assembled a chromosome-scale *L. megaphylla* genome through the integration of PacBio HiFi, Hi-C, and Illumina sequencing. The contig N50 of our high-quality assembled genome exceeded 9.09 Mb, which is significantly larger than that reported previously [15]. With access to a high-quality genome, analysis of the evolution of Lauraceae genomes and transcriptomics and metabolomics co-analysis can be applied to identify key genes involved in terpenoid biosynthesis and explore their potential applications in pest and disease control.

## Results

### Genome sequencing, assembly, and assessment of *Lindera megaphylla*

*De novo* sequencing of the *L. megaphylla* genome was conducted utilizing Hi-C, PacBio HiFi, and Illumina sequencing (Fig. 1a–d and Tables S1–S3). Initially, the K-mer approach was applied to estimate the size of the *L. megaphylla* genome, leveraging 50.2 gigabases (Gb) of data obtained from Illumina sequencing (Tables 1 and S1). The genome was estimated to span 1379.31 megabases (Mb), with a heterozygosity rate of 0.8%, as indicated in Table S1. The preliminary assembly was accomplished using both the Illumina and PacBio platforms, yielding a cumulative sequence length of 1309.08 Mb and a contig N50 of 8.82 Mb (Table S3).

**Table 1:** Statistics of *L. megaphylla* genome sequencing, assembly, and annotation

Assembly	Number
Estimated genome size by K-mer (Mb)	1379.31
Assembled genome size (Mb)	1309.20
Assembled genome size, anchored on Chr. (Mb)	1266.79
N50 of contigs (Mb)	9.09
N50 of scaffolds (Mb)	107.75
GC content (%)	39.92
Mapping rate (%)	99.79
Annotation	
Repeat sequence length (Mb)	993.77
Percentage of repeat sequences (%)	75.91
No. of predicted protein-coding genes	29 482
No. of functional annotated genes	28 657
Complete BUSCOs (%)	96.50
No. of noncoding RNAs	7004

The *L. megaphylla* genome assembly was enhanced through the application of Hi-C technology. Illumina sequencing yielded high-quality sequences totaling 179.62 Gb (Table S2). The distinct chromosomal regions were delineated using a Hi-C contact map (Fig. 1e and Table S4). The refined assembly generated a genome of 1266.80 Mb size, featuring a contig N50 and scaffold N50 of 9.08 and 107.75 Mb, respectively, as detailed in Table S5. In total, 1266.80 Mb of sequences were integrated into the 12 chromosomes of *L. megaphylla*, constituting 96.77% of the complete assembled genome (Tables S4 and S5).

The mapping rate of the reads surpassed 99.79%, suggesting that the assembly of the *L. megaphylla* genome is substantially complete, as noted in Table 1. The quality of the genome assembly was verified by the consistency quality value (QV) of Merqury, which was 43.29. The completeness and accuracy of the assembly were assessed via the LTR assembly index (LAI), which was 16.74. To evaluate the quality of the *L. megaphylla* genome and its annotations, the Benchmarking Universal Single-Copy Orthologs (BUSCO) method was utilized. BUSCO analysis revealed that 96.50% of the universal single-copy orthologs were present and complete within the *L. megaphylla* genome (Table 1).

### Genome annotation

A comprehensive analysis of the *L. megaphylla* genome revealed that approximately 75.91% of the genome sequences (993.77 Mb) were repetitive sequences (Tables 1 and S6). The majority of these repetitive sequences are long-terminal repeats (LTRs), totaling 831.65 Mb and representing 63.52% of the genome (Table S6). In terms of noncoding RNA (ncRNA), the *L. megaphylla* genome was found to contain 5261 ribosomal RNAs (rRNAs), 1397 transfer RNAs (tRNAs), 91 microRNAs (miRNAs), and 255 small nuclear RNAs (snRNAs) (Table S7). Furthermore, the genome was found to harbor 29,482 genes, of which 28,657 (97.2%) were annotated using various databases, including the nr, KEGG, InterPro, and Swiss-Prot databases (Tables S8 and S9).

### Expansion and contraction of gene families

Gene families in *L. megaphylla* were characterized and compared with those in 15 other representative species (Fig. 1f). *L. megaphylla* contained 13,675 gene families, a number lower than those found in *Pinus tabuliformis* (19,085) and *O. sativa* (13,779) but higher than those found in all other species examined, as shown in Table S10. Among *L. megaphylla* and the other 15 species, 121 single-copy gene families were detected (Table S10). Additionally, 293 species-specific gene families were uniquely detected in *L. megaphylla*. In terms of orthologs and paralogs, a comprehensive analysis revealed 1830 single-copy orthologs, 8760 multiple-copy orthologs, and 1199 unique paralogs within the *L. megaphylla* genome (Fig. 1f and Table S11).

**Figure 2:**
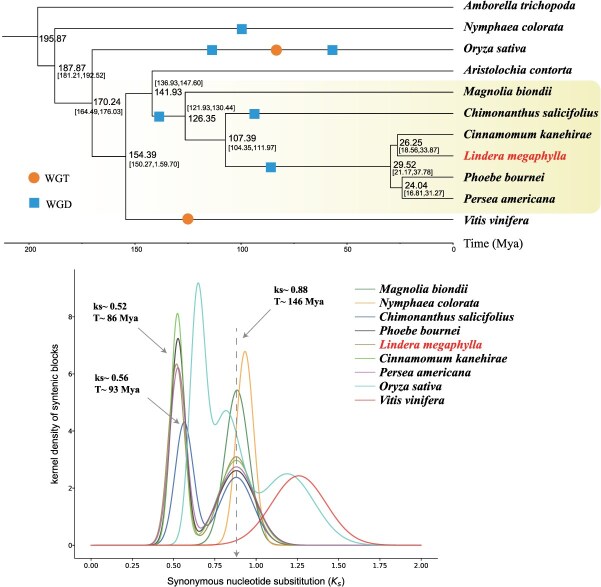
Schematic representation of the phylogenetic tree with the Ks density curve. The circles denote whole-genome triplication (WGT) events, whereas the squares indicate WGD events.

Gene family dynamics, including both contraction and expansion, were investigated in *L. megaphylla* and compared with those in 15 other representative species (Fig. 1g). In *L. megaphylla*, we identified 1677 expanded gene families, a figure that exceeds the counts observed in closely related species such as *C. kanehirae* (1067), *C. camphora* (632), and *P. bournei* (1488). Notably, *P. tabuliformis* exhibited the highest number of gene family expansions (5111), followed by *P. trichocarpa* (3853), *C. salicifolius* (3177), and *S. oleracea* (306) (Fig. 1g). The functional categories most significantly enriched in the expanded gene families of *L. megaphylla* pertain to energy metabolism and cellular growth and death, encompassing processes such as photosynthesis, oxidative phosphorylation, cell cycle regulation, and oocyte meiosis (Table S12). Conversely, *L. megaphylla* exhibited 1022 gene family contractions, more than those in *P. trichocarpa* (409) and *C. camphora* (954) but fewer than those in several other species examined (Fig. 1g). The functional categories significantly enriched in the contracted gene families of *L. megaphylla* are associated with carbohydrate metabolism and signal transduction processes, including pentose and glucuronate interconversions, as well as pathways involved in plant hormone signal transduction (Table S13).

### Phylogenetic analysis and estimation of divergence time

To explore the evolutionary narrative of *L. megaphylla*, our study included a diverse selection of 11 species, including six distinct species representing the Laurales order: four from the Lauraceae family (*L. megaphylla*, *Phoebe bournei*, *Persea americana*, and *Cinnamomum kanehirae*) one from the Calycanthaceae family (*Chimonanthus salicifolius*), and one from the Magnoliaceae family (*Magnolia biondii*) (Table S14). Additionally, we incorporated one species from the Piperales order (*Aristolochia contorta*) and one from the Nymphaeales order (*Nymphaea colorata*). To round out our study, monocotyledonous and dicotyledonous plants were represented by *Oryza sativa* and *Vitis vinifera*, respectively, with *Amborella trichopoda* representing basal angiosperms.

Initially, our construction of a phylogenetic tree elucidated the evolutionary affinities among the 11 selected species (Fig. 2). Notably, *L. megaphylla* presented the closest relationship with its congeners within the Lauraceae family. It subsequently diverged from the Calycanthaceae species *C. salicifolius* and then from the Magnoliaceae species *M. biondii*. Concurrently, we employed a series of fossil-based calibration points to anchor the divergence times of these species. This approach allowed us to deduce a more precise timeline of phylogenetic events. Building upon our initial findings, we further investigated the genomic history of these species by examining the synonymous nucleotide substitution rate, denoted as Ks. The Ks density plot revealed a distinct bimodal pattern for the five species belonging to the Lauraceae and Calycanthaceae families, which was indicative of two rounds of polyploidization. In contrast, the Magnoliaceae species *M. biondii* displayed a unimodal pattern, suggesting a singular round of polyploidization. The Piperales species *A. contorta* and the basal angiosperm *A. trichopoda* lacked any evidence of whole-genome duplication (WGD), as reflected by the absence of peaks in their Ks curves. For *L. megaphylla*, two significant WGD events were identified, with Ks values of 0.88 and 0.52 (Table S15). Drawing from the correlation between Ks values and evolutionary timelines, we estimated these events to have occurred approximately 146 and 86 million years ago, respectively (Fig. 2).

### The evolution and polyploidization of *Lindera megaphylla*

**Figure 3:**
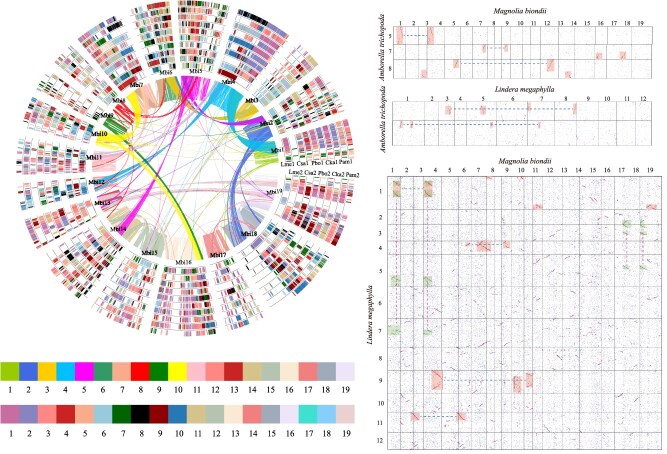
A detailed visualization of the global collinearity alignment, complemented by a partial homologous gene dot matrix. Distinct color-coded rectangles are employed to delineate and highlight the collinear regions, facilitating an intuitive understanding of the genomic correspondences among the species.

In this study, we deduced the WGD events experienced by *L. megaphylla* by examining the homology within and across genomes. This WGD event was also shared by other three species (*P. bournei*, *P. americana*, and *C. kanehirae*) of Lauraceae family (Fig. 2). Initially, when *A. trichopoda* was used as a reference, the homologous gene matrix comparison between *M. biondii* and *A. trichopoda* revealed a consistent 2:1 ratio (Fig. 3), indicative of a single WGD event in *M. biondii*, corroborated by the unimodal Ks peak of *M. biondii* (Fig. 2). By subsequently examining the homologous gene matrices between *A. trichopoda* and *L. megaphylla*, as well as those between *M. biondii* and *L. megaphylla*, we discerned clear 1:4 and 2:4 ratios, respectively (Fig. 3). Coupled with the bimodal Ks peak of *L. megaphylla* (Fig. 2), this suggests that *L. megaphylla* has undergone two rounds of WGD. Concluding our analysis, we utilized *M. biondii* as a reference to delineate the collinear regions among the five species, including *L. megaphylla*, for a comprehensive global comparison (Fig. 3 and Table S16).

### Determination of antimicrobial activities

**Figure 4:**
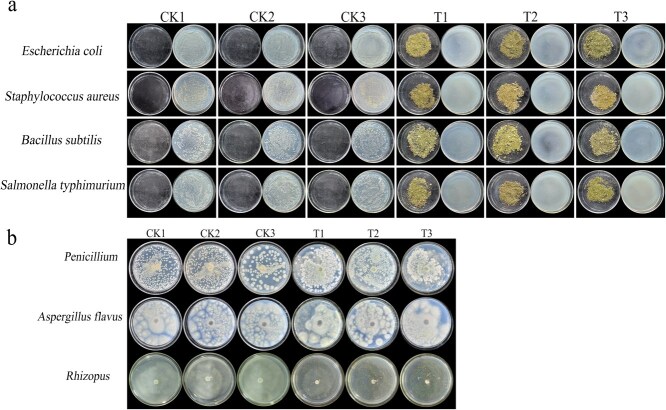
Antimicrobial activity of volatile compounds from *L. megaphylla* leaves. (a) Volatile compounds from *L. megaphylla* leaves against *E. coli*, *S. aureus*, *S. typhimurium*, and *B. subtilis.* (b) Volatile compounds from *L. megaphylla* leaves against *Penicillium*, *A. flavus*, and *Rhizopus.*

To further explore the roles of volatile compounds produced by *L. megaphylla* in promoting plant disease resistance, we first investigated the antibacterial activities of volatile compounds from *L. megaphylla* leaves. We found that the volatile compounds from *L. megaphylla* leaves significantly inhibited the proliferation of *Escherichia coli*, *Staphylococcus aureus*, *Bacillus subtilis*, and *Salmonella typhimurium*, with the inhibition rate reaching 100% when the concentrated solution was diluted 10^−6^ fold (Fig. 4a). In addition, we further investigated the roles of volatile compounds in antifungal activities and found that the volatile compounds had a significant inhibitory effect on *Rhizopus* growth but had no effect on the growth of *Penicillium* or *Aspergillus flavus* (Fig. 4b)*.* These results indicate that the volatile compounds from *L. megaphylla* have broad inhibitory effects on bacteria and species-specific inhibitory effects on fungi.

### Phylogenetic analysis of terpene synthase gene family

**Figure 5:**
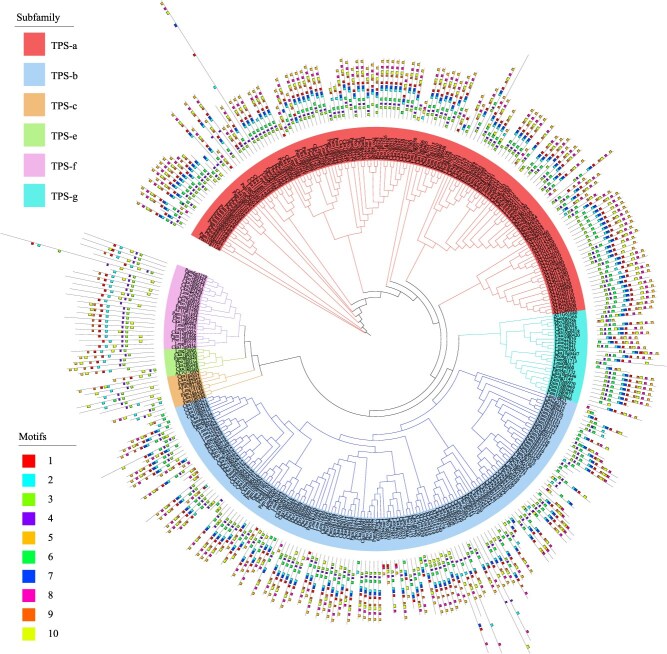
TPS family gene phylogenetic tree and conserved motif distribution. TPS-a, TPS-b, TPS-c, TPS-e, TPS-f, and TPS-g represent the 6 subfamily classifications of the TPS family. Ten different colors represent motifs 1 to 10.

Terpene synthase (*TPS*) gene family members play important roles in terpenoid biosynthesis; therefore, we identified TPS family genes from four species of the Lauraceae family (*Lindera megaphylla, Persea americana, Cinnamomum kanehirae, Phoebe bournei*) and *Arabidopsis thaliana*. After a rigorous screening process, we confirmed a total of 330 *TPS* genes across the five species, with 33 genes in *A. thaliana*, 52 genes in *L. megaphylla*, 75 genes in *P. americana*, 86 genes in *P. bournei*, and 84 genes in *C. kanehirae*. Based on the phylogenetic tree topology and the known classification of *A. thaliana TPS* genes, we categorized *L. megaphylla TPS* genes into six distinct groups (Fig. 5). The TPS-a subfamily contained the most genes, with 23 genes from *A. thaliana*, 19 genes from *L. megaphylla*, and the highest number, 37 genes, from *P. bournei*. The TPS-b subfamily had the second-highest number of genes, with 6 genes from *A. thaliana*, 20 genes from *L. megaphylla*, and the highest number, 41 genes, from *C. kanehirae*. The TPS-c, TPS-e, TPS-f, and TPS-g subfamilies contained relatively fewer genes overall. Compared with *A. thaliana*, the number of *TPS* genes in the TPS-b, TPS-f, and TPS-g subfamilies in *L. megaphylla* was significantly increased*.* In plants, TPS-b is responsible for catalyzing the formation of monoterpene compounds; TPS-f plays a role in the synthesis of *ent*-kaurene as well as other monoterpene, sesquiterpene, and diterpene compounds; and TPS-g is specialized in the synthesis of specific terpenes that contribute to the flavor and aroma of ripe fruits [25–28]. Therefore, these three types of TPS family genes may play important roles and be related to specific terpene synthesis in *L. megaphylla.* Importantly, the TPS-d group lacks *A. thaliana* counterparts and thus, was not included in our analysis [25, 29]. Additionally, we performed a detailed analysis of the conserved motifs. The motif distribution patterns indicate that genes within the same subfamily present a high degree of similarity in their domain architecture, suggesting strong conservation across the *TPS* gene family (Fig. 5).

### Transcriptomic and metabolomic analyses in different tissues of *L. megaphylla*

**Figure 6:**
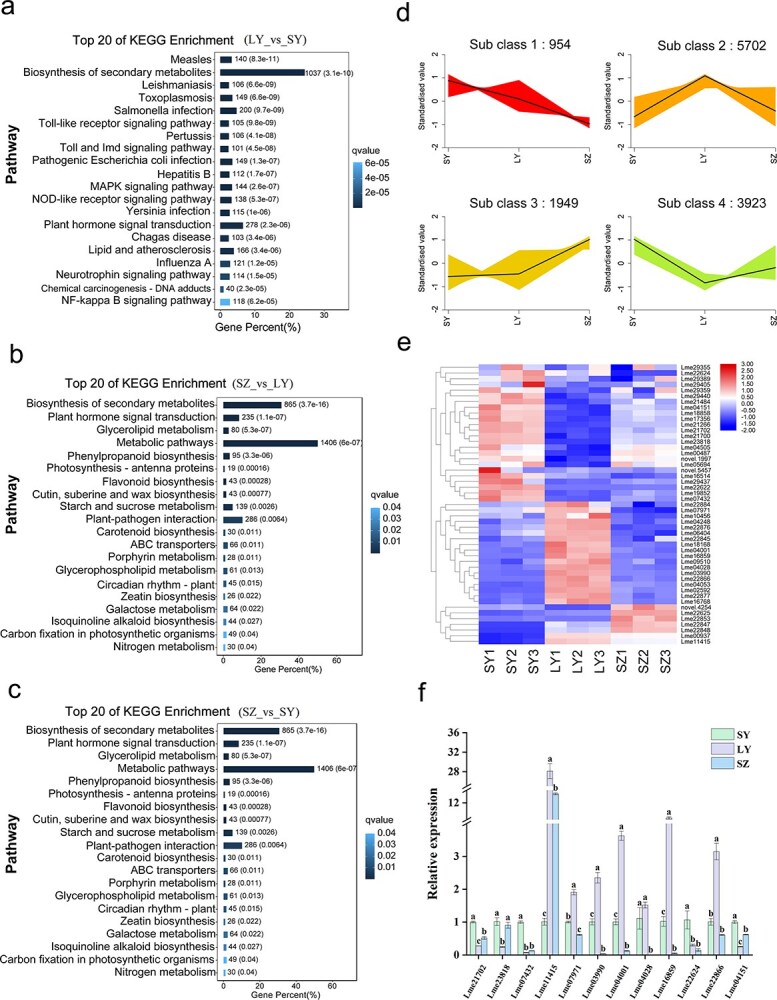
Identification of DEGs in different comparison groups*.* (a-c) KEGG enrichment analysis of DEGs in the comparisons of LY vs. SY, SZ vs. LY, and SZ vs. SY. (d) Module analysis of DEGs. The *Y*- and *X*-axes indicate the *P* values and tissues, respectively. (e) A heatmap was constructed to display the expression of DEGs associated with terpenoid synthesis based on RNA-seq data. (f) RT-qPCR verification of 12 candidate genes associated with terpenoid synthesis. The error bars reflect the standard error of three replicates, indicating the variability in the expression data. Note: SY, LY, and SZ represent leaves, leaf buds, and annual shoots, respectively.

Combination of transcriptomic and metabolomic analyses was used to investigate the dynamic changes of genes and metabolites in different tissues of *L. megaphylla* (Figs S1–S3 and Table S17). A total of 10,079 differentially expressed genes (DEGs) were detected in the comparison of leaf buds (LY) versus leaves (SY), including 5390 upregulated and 4689 downregulated DEGs (Table S18), which were further functionally annotated by integrating GO function and KEGG pathway enrichment analyses (Fig. 6a, Fig. S4a, and Tables S19 and S20). In the comparison of annual shoots (SZ) versus LY, identification of 7842 DEGs including 3343 upregulated and 4499 downregulated DEGs (Table S21) were used to investigate the potential functions using GO function and KEGG pathway enrichment analyses (Fig. 6b, Fig. S4b, and Tables S22 and S23). In the comparison between SZ and SY, a total of 5968 DEGs (2784 upregulated and 3184 downregulated) (Table S24) were annotated using the GO function and KEGG pathway enrichment analyses (Fig. 6c, Fig. S4c, and Tables S25 and S26). The K-means clustering analysis of DEGs was performed to generate four co-expression clusters (Fig. 6d). The result revealed that the DEGs in the same cluster showed similar expression patterns, indicating that these DEGs might have specific functions in different tissues. Additionally, forty-nine DEGs involved in terpenoid biosynthesis were identified in the three comparison groups (Fig. 6e and Table S27)*.* Further analysis revealed that twenty-four DEGs (15 *GFPS*s, 3 *TPS*s, 2 *DXS*s, 2 *HDS*s, 1 *DXR*, and 1 *AACT*) were significantly upregulated in the comparison of LY versus SY (Table S28). In the comparison of SZ versus LY, transcripts of *LmTPS1* (Lme04028) from the TPS-a group increased 64-fold, and other terpenoid biosynthesis-related genes, including *DXS* (Lme18168), *GFPSs* (Lme02592 and Lme06404), and *MVK* (Lme04248), presented similar trends (Table S29). Fourteen DEGs, including seven *GFPSs*, 3 *TPSs*, 2 *DXS*, and 2 *HDSs*, were significantly upregulated in the comparison of SZ versus SY (Table S30). To further confirm the reliability of the RNA-seq data, we performed RT-qPCR to verify the expression of 12 selected DEGs involved in terpenoid biosynthesis (Figs 6f and  S5). The results demonstrated the high accuracy and reliability of the RNA-seq results.

**Figure 7:**
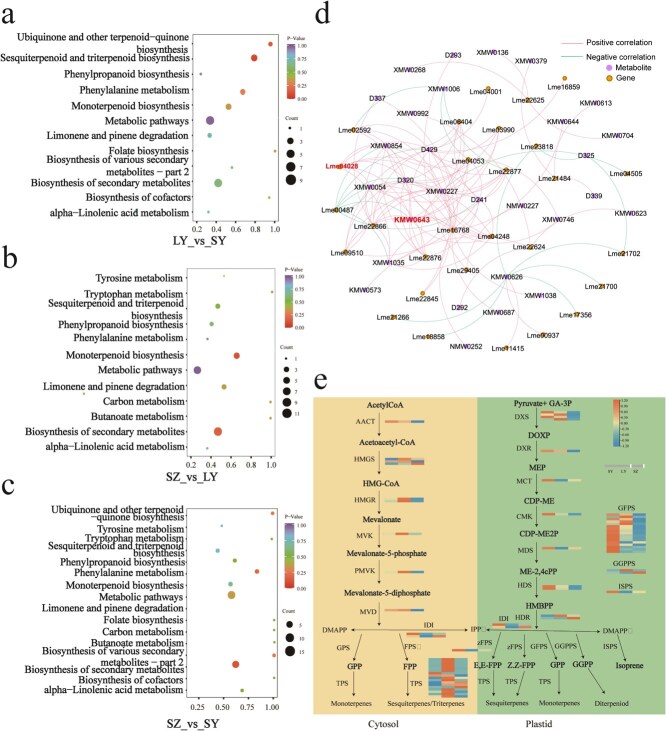
Transcriptomic and metabolomic co-analysis of DEGs and DEMs involved in the terpenoid biosynthesis pathway. (a-c) KEGG enrichment analysis of DEMs in the comparisons of LY vs. SY, SZ vs. LY, and SZ vs. SY. (d) Correlation analysis was conducted between a subset of DEGs and DEMs related to terpenoid synthesis in *L. megaphylla*. (e) Tissue-specific relative expression profiles of DEGs implicated in terpenoid biosynthesis. The red and blue boxes indicate the up- and down-regulated DEGs, respectively, in different tissues of *L. megaphylla*. Cytosol represents the MVA pathway, and plastid represents the MEP pathway.

Metabolomics analysis showed that 387 differentially expressed metabolites (DEMs), consisting of 236 metabolites with increased concentration and 151 metabolites with decreased concentration were detected in LY versus SY (Table S31), 302 DEMs (214 upregulated and 88 downregulated metabolites) in SZ versus LY (Table S32), and 321 DEMs (259 upregulated and 62 downregulated metabolites) in the comparison between SZ and SY (Table S33). KEGG enrichment analysis was then performed to analyze their biological functions of these DEMs form the three comparison groups (Fig. 7a–c).

KEGG enrichment analysis of these DEGs and DEMs revealed significant enrichment in pathways such as secondary metabolite synthesis (ko01110), sesquiterpenoid and triterpenoid biosynthesis (ko00909), monoterpenoid biosynthesis (ko00902), and limonene and pinene degradation (ko00903) (Fig. 6a–c, Fig. 7a–c, and Tables S20, S23, and  S26). Intergroup correlation networks of DEMs and DEGs enriched in terpenoid biosynthesis pathway were constructed. A correlation value > |0.85| and a *P* value <.05 were used as the criteria to screen DEGs highly correlated with DEMs. The results revealed that 43 DEGs were strongly correlated with 117 terpenoid compounds (Fig. 7d and e and Table S34)*.* Further analysis revealed that thirteen DEGs, including 9 *TPSs*, 3 *GGPPSs*, and 1 *MVK*, were positively correlated with the content of caryophyllene oxide, demonstrating that these DEGs could participate in regulating the biosynthesis of caryophyllene oxide. Among them, the *LmTPS1* (Lme04028) gene was significantly related to the content of caryophyllene oxide (Table S34 and Fig. S6).

### Molecular cloning and bioinformatic analysis of *LmTPS1*

**Figure 8:**
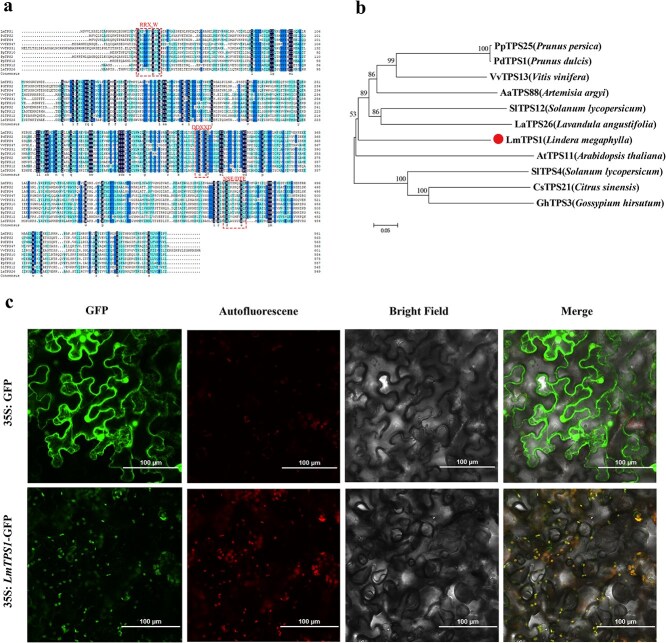
Protein sequence alignment, phylogenetic analysis, and subcellular localization of LmTPS1. (a) Multiple sequence alignment of LmTPS1 and homologous proteins from other plants. Conserved motifs are highlighted in dashed rectangle. (b) Phylogenetic analysis showing the evolutionary relationships between LmTPS1 and homologous proteins from other plants. (c) Subcellular localization of the LmTPS1 protein in tobacco leaves. GFP alone or the *LmTPS1* gene fused with GFP driven by the CaMV35S promoter was transiently expressed in tobacco epidermis cells. Transgenic plants expressing candidate genes fused to GFP were detected by laser scanning confocal microscopy. Scale bar: 100 μm.

To further reveal the gene function of *LmTPS1*, we first cloned the full length coding sequence of *LmTPS1* gene, and the results showed that the cloned *LmTPS1* contained an open reading frame of 1683 bp encoding 561 amino acids, which had a conserved DDxxD and NSE/DTE motifs (Figs 8a and  S7). Phylogenetic analysis revealed that the *L. megaphylla LmTPS1* was clustered closely with the identified *LaTPS26* and *SlTPS12* involved in β-caryophyllene biosynthesis (Fig. 8b), indicating that *LmTPS1* might participate in regulating the biosynthesis of β-caryophyllene in *L. megaphylla*.

We determined the subcellular localization of the LmTPS1 protein in tobacco leaf cells via transient transformation (Fig. 8c). The results revealed that the LmTPS1 protein was localized in the chloroplasts and cytoplasm, whereas free GFP was detectable throughout the cells. These findings indicate that the LmTPS1 protein might participate in the biosynthesis of terpenoid compounds through both the MEP and MVA pathways.

### Overexpression of *LmTPS1* improved the resistance of transgenic tomato to biotic stress

**Figure 9:**
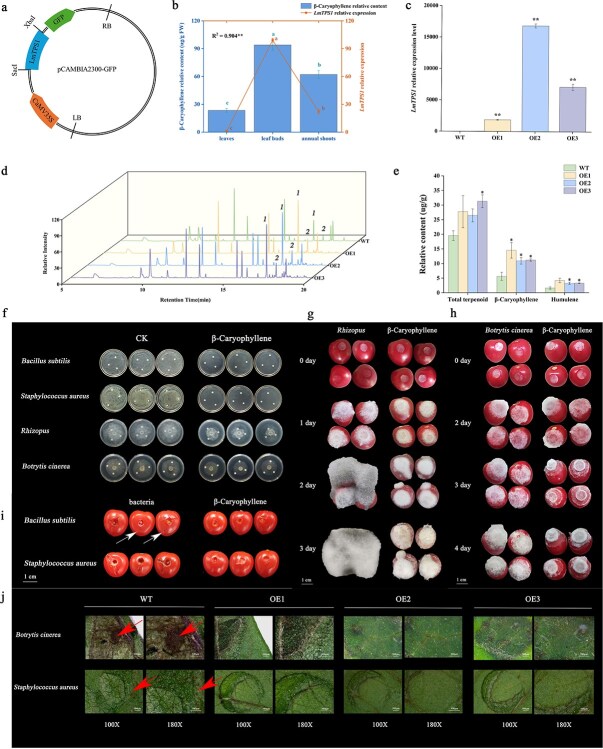
Overexpression of *LmTPS1* improved the resistance of transgenic tomato to biotic stress. (a) Structural diagram of *LmTPS1*-pCAMBIA2300. (b) Tissue-specific correlation analysis between *LmTPS1* expression and β-Caryophyllene accumulation in *L. megaphylla*. (c) RT-qPCR verification of *LmTPS1 in* transgenic tomato lines (OE1-OE3). (d) Measurement of terpenoids in transgenic tomato lines and WT plants using GC–MS/MS. (e) The contents of three main terpenoids in the transgenic tomato lines. (f) The inhibitory effects of β-caryophyllene on *S. aureus*, *E. coli*, *S. typhimurium*, and *B. subtilis.* (i-h) Tomato fruits injected with β-caryophyllene presented increased resistance to biotic stress. (j) Overexpression of *LmTPS1* improved the resistance of transgenic tomato to biotic stress. The leaf lesion areas were captured under 100x and 180x magnification using a super depth-of-field microscope, with arrows indicating the diseased regions on the leaf. Scale bar: 500 μm.

To further verify the role of the *LmTPS1* gene in the biosynthesis of β-caryophyllene and related compounds, the expression levels of *LmTPS1* in three different tissues, namely, the leaves (SY), leaf buds (LY), and annual shoots (SZ), were investigated using RT-qPCR, which revealed a strong correlation (*R*^2^ = 0.904) with the β-caryophyllene content (Fig. 9b). Thus, we generated seven transgenic tomato lines overexpressing the *LmTPS1* gene using the Agrobacterium-mediated transformation method to investigate the functions of *LmTPS1* in the biotic stress response (Fig. S8a and b). Three independent *LmTPS1*-overexpressing tomato lines (OE1, OE2, and OE3) were selected and used to investigate the function of the *LmTPS1* gene in terpenoid biosynthesis based on kanamycin-resistance and RT-qPCR detection (Fig. 9a and c). Seven-week-old transgenic tomato plants were used to measure the contents and types of terpenoids. The results revealed that the total content of terpenoid compounds increased compared with that in the control group. Among them, the contents of two terpenoids (β-caryophyllene and humulene) in the transgenic tomato lines were found to exceed those in WT plants by more than two-fold (Fig. 9d and e). These results indicated that the *LmTPS1* gene was involved mainly in regulating the biosynthesis of β-caryophyllene and humulene. β-Caryophyllene and humulene play important roles in resisting foreign invading microorganisms, which are the main aroma components of *L. megaphylla.* Compared with the control group, a medium containing 0.9 g/mL β-caryophyllene completely inhibited the growth of *B. subtilis* and *S. aureus*, and the average hyphae of *B. cinerea* and *Rhizopus* grown in medium containing 0.9 g/mL β-caryophyllene were approximately 4.81 and 5.22 cm, respectively, significantly smaller than those grown in the blank medium (5.97 and 7.59 cm, respectively) (Fig. 9f). Moreover, β-caryophyllene could reduce the degree of tomato fruit decay caused by *B. subtilis* and *S. aureus* (Fig. 9i), which also inhibited the growth rate of *Rhizopus* and *B. cinerea* disease on the surfaces of the tomato fruits (Fig. 9g and h). In addition, the resistance of transgenic tomato lines overexpressing the *LmTPS1* gene to *B. cinerea* and *S. aureus* was explored through inoculation with cell suspensions, and the results revealed that the dark lesions caused by *S. aureus* and the gray mold disease caused by *B. cinerea* on the surfaces of leaves from the transgenic tomato lines were significantly less severe than those on the surfaces of leaves from the WT plants (Fig. 9j).

## Discussion

*L. megaphylla*, a member of the Lauraceae family, is part of the Laurales order. The genomes of several species within this order have been sequenced and made publicly available, including 2 species from the Calycanthaceae family and 11 from the Lauraceae family (https://www.plabipd.de/pubplant_cladogram1.html). The genome quality we report is superior to that of the previously reported *L. megaphylla* genome [15]. For example, the contig N50 of our assembled genome is 9.09 Mb, which is much larger than the contig N50 (2.61 Mb) of the previously reported genome. Moreover, the BUSCO score (96.50%) of our assembled genome was also higher than that (91.70%) of the previously reported genome. This high-quality *L. megaphylla* genome will contribute to comparative genomic analyses in Lauraceae and other families of the order Laurales.

During the assembly process, several challenges were encountered, especially in integrating PacBio HiFi, Hi-C, and Illumina data. The different data types possess varying error profiles and read lengths, making alignment and merging complex. PacBio HiFi offers long, accurate reads but at a relatively high cost and low throughput. Hi-C data help with scaffolding but require careful handling to avoid misjoins. Illumina data have high accuracy but shorter read lengths. The current genome version, despite achieving a relatively high-quality chromosome-level assembly, still falls short of the T2T (telomere-to-telomere) level. Gaps remain in centromeric and telomeric regions, and complex repetitive sequences are not fully resolved. This limits our understanding of genomic functions and evolutionary processes precisely in these critical areas, highlighting the need for further advancements in sequencing and assembly techniques.

Phylogenetic analysis based on chloroplast genomes has shown that *Lindera* species can be divided into two subclades, with *L. megaphylla* being part of subclade II, which is well supported by bootstrap analysis [24, 30]. This subclade includes species such as *Laurus nobilis* and *Litsea glutinosa*, indicating a close relationship with *L. megaphylla* [24]. Phylogenetic studies have placed Lauraceae, and by extension *L. megaphylla*, in a sister relationship to eudicots after their divergence from monocots [15]. This suggests a complex evolutionary history within the Laurales order. Among the species we selected, *L. megaphylla* was found to be evolutionarily more closely related to *C. kanehirae*, *C. camphora*, and *P. bournei*. In summary, the high-quality *L. megaphylla* genome obtained in this study provides important genomic data resources for subsequent evolutionary studies of species in the Lauraceae and even the order Laurales.

*L. megaphylla*, a traditional Chinese medicinal plant, is used for the treatment of anxiety and depression due to its anesthetic and anti-inflammatory properties [20–22]. Previous studies have reported that nonvolatile medicinal ingredients (e.g. D-dicentrine, (+)-dicentrine, and northalifoline) in *L. megaphylla* have antitumor, antiplatelet aggregation, vasorelaxant, and antiarrhythmic activities [22]. However, very little research has been conducted on its volatile medicinal ingredients. Through metabolomics analysis, we identified a total of 534 DEMs, including 134 terpenoid compounds, which are used in seasonings (camphene, β-pinene, α-pinene, fenchone, and ledol), cosmetics (e.g. eucalyptol, citronellal, citronellol, and carveol), and pharmaceuticals (e.g. caryophyllene oxide, germacrene D, (−)-spathulenol, and terpinen-4-ol). Natural antimicrobials from plants are widely applied to protect food from pathogenic and spoilage microorganisms [31]. Caryophyllene (β-caryophyllene) is a major sesquiterpene with biological properties, including pathogen resistance, antiherbivory, and chemical signals for plant-pollinator interactions [12, 32, 33]. In addition, volatile β-caryophyllene has been widely applied in the pharmaceutical, cosmetic, and food industries [34–36]. Numerous studies have revealed that caryophyllene biosynthesis is catalyzed by *TPS* genes using FPP as a substrate [12, 14, 37]. However, the molecular mechanism underlying β-caryophyllene biosynthesis in *L. megaphylla* remains unclear. Which *TPS* gene is involved in regulating β-caryophyllene biosynthesis? Due to the lack of an efficient and stable genetic transformation system in *L. megaphylla*, tomato was selected as a model plants to verify the roles of *LmTPS1* due to its efficient and stable genetic transformation system, shared terpenoid biosynthesis pathways with *L. megaphylla*, especially sesquiterpene, and emerged as the model organism of choice for research for plant-pathogen interactions [26]. In the present study, we investigated the candidate *TPS* genes responsible for the production of various terpenoids in different tissues of *L. megaphylla*. RNA-seq of the three different tissues identified 18 DEG *TPS* gene*s* out of 43 DEGs associated with the terpenoid biosynthesis pathway, indicating that the 18 DEG *TPS*s identified play essential roles in regulating terpenoid biosynthesis in the *L. megaphylla* studied here. Transcriptomic and metabolomic co-analysis revealed that nine *LmTPS* genes, especially *LmTPS1* (Lme04028), were positively correlated with the content of caryophyllene oxide, indicating that these genes might be involved in caryophyllene oxide biosynthesis, which is consistent with the findings of previous studies indicating that *TPS*s could contribute to the regulation of caryophyllene biosynthesis. For example, *Arabidopsis AtTPS11* and *AtTPS21* are involved in regulating caryophyllene biosynthesis [38]. In this study, seven transgenic tomato lines overexpressing *LmTPS1* were generated and validated via PCR and RT-qPCR confirming successful *LmTPS1* integration. We also investigated whether the *LmTPS1* gene was crucial in volatile caryophyllene biosynthesis in transgenic tomato plants. A total of fifteen differentially accumulated volatile terpenoids were detected in transgenic tomato and WT plants. Among them, the contents of four terpenoids (β-elemene, β-caryophyllene, humulene, and γ-selinene) in transgenic tomato lines was higher than those in WT plants. Notably, the contents of β-caryophyllene and its homologous isomers (Humulene) in transgenic tomato lines were at least twice higher than those in WT plants. The contents of β-elemene and γ-selinene in transgenic tomato lines were slightly higher than those in WT plants. These results revealed that *LmTPS1* mainly contributed to promote the accumulation of β-caryophyllene and its homologous isomers (Humulene) in transgenic tomato plants. Moreover, pathogen inoculation experiments have further revealed that β-caryophyllene, which is catalyzed by *LmTPS1*, enhances the defense response of transgenic tomato lines against *B. cinerea* and *S. aureus*, which is consistent with the conclusion of previous research that β-caryophyllene contributes to increased pathogen resistance [25, 29]. β-Caryophyllene has demonstrated potent antimicrobial activity against a variety of bacteria and fungi, making it a promising candidate for developing new antibiotics and antifungal agents, especially amid increasing resistance to existing drugs. Further investigations into the specific mechanisms of the antimicrobial action of β-caryophyllene could lead to the design of more targeted and effective therapeutics. Additionally, the preservative effect of β-caryophyllene is beneficial for improving postharvest storage, as it helps extend the shelf-life of fruits and vegetables. This can lead to reduced postharvest losses and increased economic benefits for agricultural production. β-Caryophyllene also has anti-inflammatory properties, which are crucial for the treatment of a broad spectrum of inflammatory diseases, including arthritis and inflammatory bowel disease. Thus, the discovery of β-caryophyllene biosynthesis, which is catalyzed by *LmTPS1* in transgenic tomato lines, not only provides new insights into crop improvement but also has significant implications for medicinal research. By harnessing the antimicrobial and anti-inflammatory potential of β-caryophyllene and related compounds, researchers can explore innovative approaches for developing effective therapeutics and sustainable agricultural practices that benefit human health.

## Conclusion

In this study, we assembled a high-quality chromosomal-scale *L. megaphylla* genome. With a total length of 1309.2 Mb, the genome encompasses 29,482 annotated genes. Our analysis revealed two WGD events within the evolutionary history of the *L. megaphylla* genome. A total of 52 TPS family genes were found in *L. megaphylla*, and the number of *TPS* genes in the three groups TPS-b, TPS-f, and TPS-g was significantly expanded. Transcriptomic and metabolomic co-analysis revealed that the expression levels of nine *TPS* genes, especially *LmTPS1*, were strongly correlated with the content of caryophyllene oxide. The overexpression of *LmTPS1* in transgenic tomato significantly increased the contents of β-caryophyllene and humulene, which could be further used to improve the resistance of common fungal and bacterial diseases. This analysis provides insights into the evolution of terpenoids in *L. megaphylla* and elucidates the molecular mechanisms underlying their protective effects. These findings, coupled with the results of the comparative genomic analysis, provide a wealth of information that will help us understand the gene function and evolutionary dynamics of *L. megaphylla* and related plant species.

## Materials and methods

### Genome sequencing and genome size estimation

The leaves of male *L. megaphylla* were sampled from Huiche town (33°28′01″ N, 111°06′10″ E), Xixia County, Henan Province, China, in May 2022. The isolation of genomic DNA from *L. megaphylla* leaves was performed using a QIAGEN kit following the standard protocol. The quantification and purity of the isolated DNA were measured using a Qubit® 3.0 fluorometer and a NanoDrop™ One spectrophotometer, respectively. The genomic libraries were sequenced on both the PacBio and Illumina platforms, adhering to established methods [39, 40]. Furthermore, integrated technologies of Hi-C and Illumina sequencing improved the genome assembly. The size of the *L. megaphylla* genome was estimated utilizing the K-mer approach, as referenced in previous studies [41, 42].

### Quality control and genome assembly

Quality control (QC) and genome assembly procedures were conducted using established bioinformatics pipelines. For third-generation sequencing data processing, SMRT Link software was employed to perform initial quality control, during which adapter sequences were identified and removed to generate refined subreads. These subreads were subsequently processed through the Circular Consensus Sequencing (CCS) module with stringent parameters: a minimum of three full passes and a read quality score threshold of ≥0.99, to produce high-fidelity (HiFi) reads meeting the required accuracy standards.

The genome assembly of *L. megaphylla* was constructed using the Hifiasm program through a systematic three-phase approach [43]. In the initial error correction phase, the software performed comprehensive error correction and all-by-all pairwise comparisons using the complete set of HiFi reads. The second phase involved constructing a phased assembly string graph that preserved haplotype information through overlap-based graph construction. Finally, the assembly process was completed by resolving graph complexities through bubble detection and selecting optimal contig paths, thereby generating high-confidence chromosomal-level scaffolds.

### Hi-C data-assisted assembly and genome assessment

The QC of Hi-C data encompasses alignment verification and HiCUP-based quality assessments [44]. Employing Hi-C technology, the ALLHiC algorithm was utilized to assemble the genome according to previous reports [45, 46]. Juicebox software was used for visualization of the clustered BAM files and genomes generated by ALLHiC [47]. The completeness and accuracy of the assembled genome were subsequently evaluated using BUSCO software with the embryophyta_odb10 database [48, 49]. Additionally, the BWA software was applied to align the assembled genomes and NGS data [50]. The completeness and accuracy of the assembly were assessed using the LTR assembly index (LAI) [51]. The quality of the genome assembly was also verified by the consistency quality value (QV) of Merqury (https://github.com/marbl/merqury).

### Genome annotation

Integrated homologous alignment and *de novo* prediction were used to detect the repeated sequences. Initially, a comprehensive repeat sequence database was constructed using RepeatModeler, LTR_FINDER [52], RepeatScout [53], and PILER [54]. Subsequently, homologous sequence searches were conducted using the RepeatMasker and RepeatProteinMask programs, which search the Repbase database [55, 56]. Additionally, INFERNAL software was used to identify miRNAs and snRNAs [57]. tRNA and rRNAs were detected using tRNAscan-SE and BLAST, respectively [58].

### Prediction of protein-coding genes and functional annotation

*De novo* gene prediction was performed using GlimmerHMM [59], SNAP [60], and Augustus. Homologous genes were identified using BLAST and GeneWise [61, 62]. The results from these predictions were consolidated using the IntegrationModeler (EVM) pipeline [63]. The gene predictions from EVM were subsequently refined by combining the RNA-seq data using PASA software [64]. The genes were annotated using the protein databases KEGG, TrEMBL, InterPro, and Swiss-Prot.

### Identification of gene families and expansion/contraction analysis

OrthoFinder software was used to detect gene families in *L. megaphylla* and other representative plant genomes obtained from the PlantGIR database [65, 66]. The process began with filtering for alternative splicing events within each species. Proteins less than 100 aa long were subsequently removed. Comprehensive all-by-all BLAST analyses were performed by comparing all protein sequences among the investigated species with a stringent E value threshold of less than 1e-5. Cluster analysis was then performed to detect both single- and multicopy gene families using the MCL algorithm with an inflation parameter set to 1.5. CAFE software was used to explore the expansion/contraction of gene families, applying a significance threshold of 0.05, a tree topology of 4, and a randomization count of 10 000 [67].

### Phylogeny analysis and divergence time estimation

In our research, we employed the OrthoFinder (v2.0) [65] software suite, leveraging its capabilities for multiple sequence alignment (−M msa) and expedited alignment (-S diamond), to meticulously identify single- or low-copy genes that served as the cornerstone for our phylogenetic analysis. We then proceeded to align the concatenated amino acid sequences with precision using MAFFT [68] and refined the alignments using the trimAI [69] tool to increase the quality of our sequence data. Using RAxML [70], we constructed a maximum likelihood phylogenetic tree under the PROTGAMMAJTT model, substantiated by 1000 bootstrap replicates, with *Amborella trichopoda* strategically positioned as the outgroup. To refine the divergence time estimates, we integrated our species tree into the MCMCTree program of the PAML [71] package. The temporal calibration was meticulously anchored using a series of fossil records sourced from the TimeTree [72] database, thereby reinforcing the precision and dependability of our study’s conclusions.

### Gene collinearity detection and visualization

In this research, we conducted an in-depth evaluation of genomic collinearity using WGDI [73] software according to previous reports [74, 75]. We commenced by pinpointing homologous genes across the genomes of two species using the BLASTP tool (E value ≤1e^−5^) [28]. We then using the ‘–icl’ model for collinearity detection, with the maximal gap length set to 50, and meticulously excluded over 30 gene families to broaden our detection capabilities. The WGDI program was subsequently utilized to construct dot plots to visually represent collinear genes. Concluding our analysis, we established collinear alignments for Laurales species, referencing the MBI genome. Given the occurrence of two WGD events, each Laurales species is theorized to correspond to two columns. The ‘–ci’ parameter of the WGDI program was skillfully applied to render a circular visualization [73], offering an intuitive and graphical representation of genomic collinearity. The synonymous substitution rate (Ks) typically does not affect the composition of amino acids and is not influenced by natural selection. Consequently, the distribution of Ks is often used as a basis for determining polyploidization events [76]. We calibrated the Ks values for the selected magnoliid species. The relationship between polyploidization time and Ks can be expressed as *T* = Ks/2r, where r is the evolutionary rate of 3.02 × 10^−9^ for the Magnoliaceae family [77].

### Ks calculation and distribution fitting

In this research, we initially used the MUSCLE [78] program to align homologous amino acid sequences. We then used the PAL2NAL [79] program to translate the protein alignments into codon alignments in accordance with the coding DNA sequences (CDSs). The Ka and Ks values were subsequently calculated using the yn00 program within the PAML [71] package. Within collinear blocks, segments resulting from gene duplication events were categorized based on the median Ks values among homologous genes. The distribution of Ks values across these blocks was vividly represented in the WGDI [73] tool, differentiated by various colors. The profiling of the Ks density distribution was meticulously conducted through three distinct modules of the WGDI suite: (i) the Ks density distribution curve was delineated using the KsPeaks module with the (−kp) option; (ii) the PeaksFit module, denoted by (−pf), was applied to carry out multipeak fitting; and (iii) the KsFigures module, indicated by (−kf), was used to amalgamate the various fitted density curves into a unified graphical representation, thereby providing a clear visual depiction of the Ks distribution patterns.

### Nontargeted GC–MS metabolomic profiling analysis

Three different tissue samples, namely, leaves (SY), leaf buds (LY) and annual shoots (SZ), were collected from *L. megaphylla* and immediately frozen in liquid nitrogen. Volatile components were detected by Wuhan Metware Biotechnology Co., Ltd. (Wuhan, China) using GC–MS/MS (Agilent Technologies Inc.). In total, 1 g powder was placed into a 20-mL headspace vial with 2 ml of NaCl saturated solution. Each vial was incubated at 60°C for 5 min, followed by 15 min of exposure of a 120-μm DVB/CAR/PDMS fiber (Agilent) to the sample headspace at 100°C for SPME analysis. GC–MS conditions: the volatile components were desorbed from the fiber by inserting the fiber into the injection port of an Agilent gas chromatograph. The volatile components were detected using a DB-5 ms column (30 m × 0.25 mm) housed in an Agilent 8890 gas chromatograph with an Agilent 7000D mass spectrometer. Helium (He) was used as the carrier gas, with a flow rate of 1.2 mL/min. The injector and detector temperatures were set at 250 and 280°C, respectively. The oven temperature program was as follows: 40°C (3.5 min), +10°C/min to 100°C, +7°C/min to 180°C, and + 25°C/min to 280°C (held for 5 min). MS was performed in EI mode at 70 eV; the quadrupole, ion source, and transfer line were set at 150, 230, and 280°C, respectively. SIM mode was used for analyte ID and quantitation. A VIP value ≥1 and log_2_(fold change) ≥1 were used as screening criteria to identify the significantly different VOCs between groups.

### Transcriptomic analysis

Leaves (SY), buds (LY), and annual shoots (SZ) from *L. megaphylla* were sampled and stored in liquid nitrogen. The extraction and detection of total RNA were conducted as described previously [23]. For each sample, which included three biological replicates, 1 μg of total RNA was submitted to Wuhan Metware Biotechnology Co., Ltd., for RNA-Seq. After low-quality reads and adapters were removed, the clean reads were assembled using Trinity software [80]. Gene abundances were normalized by calculating the fragments per kilobase of exon model per million reads mapped value (FPKM) to account for variation in gene length. Six databases (NR, KEGG, GO, Pfam, Swiss-Prot, and eggNOG) were used to annotate the unigenes. RSEM software was used to calculate unigene expression levels using the FPKM method. The correlation coefficient (*r*) was used to assess the correlation between expression profiles across samples. Principal component analysis (PCA) was applied to group the samples in each treatment group based on the expression profiles of the unigenes. Pairwise DEGs were identified using the DESeq2-R package with a threshold of log_2_(fold change, FC) ≥ 1 and a *q* value <0.05. A heatmap was generated to visualize the transcript abundance of the DEGs. Moreover, GOseq from the R package and the KOBAS (v2.0) web server were used to conduct GO analysis and KEGG enrichment analysis of the DEGs, respectively [81].

### Co-expression network analysis

In the R environment, Pearson’s correlation coefficients were applied to build the interaction network based on the correlation coefficients of |R| ≥ 0.9 and *P* value ≤0 .05. Co-expressed genes with strong interconnectivity were identified as hub genes. To visualize the relationships between DEGs and terpenoid components, Gephi was used to construct a correlation network diagram.

### Identification and analysis of *TPS* genes

First, we obtained HMM models for the *TPS* gene family (PFAM IDs: PF03936 and PF01397) from the Pfam database [27, 82]. We then used the hmmsearch tool to search the target protein sequence database, and the members of the *TPS* gene family retrieved became candidate genes for the family. Based on the E value, we performed a selection to ultimately identify TPS family genes according to previous reports [27, 28, 83, 84]. Furthermore, we used the MAFFT method [85] to align the TPS family genes and then constructed a phylogenetic tree using the FastTree tool [86]. We then used the MEME tool [87] to analyze the conserved motifs within the *TPS* gene family, setting the number of motifs to 10, with a minimum width of 6 and a maximum width of 50, while keeping all other parameters at their default values [88, 89]. Finally, we employed iTOL software [90] to visualize the phylogenetic tree of the TPS family genes along with the conserved motifs, as described in previous reports [91, 92].

### Real-time PCR validation

The transcript abundances of the 12 DEGs obtained from the RNA-seq of three different *L. megaphylla* tissues were verified using real-time PCR (RT-qPCR). *UBC28* was selected as a reference gene in RT-qPCR analysis to investigate the transcript abundance of 12 candidates via the 2^−ΔΔCT^ method [93, 94]. Each experiment was performed with three biological and technical replicates. The primers used are listed in Table S35.

### Gene cloning and subcellular localization

The *LmTPS1* coding sequence was amplified using cDNA templates extracted from *L. megaphylla* leaves. Structural domain analysis was performed using the SMART online tool (http://smart.embl-heidelberg.de/). Amino acid sequence alignment and comparative analysis were executed with DNAMAN software (v9.0). Phylogenetic relationships analysis between *LmTPS*1 and functionally characterized homologous proteins from other species was conducted using MEGA software (v11.0) with the neighbor-joining method.). All protein sequences were retrieved from the NCBI GenBank database for comparative analysis.

The coding sequence of the *LmTPS1* gene was cloned and inserted into the pCAMBIA2300-GFP vector. *A. tumefaciens* (GV3101) transformed with the binary vectors was transiently transformed into tobacco leaf epidermis cells*.* The fluorescence signal in tobacco leaves was detected after 2 d of incubation by confocal laser scanning microscopy (Leica TCS SP5-II, Leica Microsystems, Wetzlar, Germany) [95].

### Antibacterial and antifungal activities of volatile compounds

The antibacterial activities of volatile compounds were measured using previously reported methods with slight modifications [96]. Nutrient agar medium was prepared (5 g of beef extract, 10 g of peptone, and 5 g of NaCl, pH 7.0) with an additional 20 g of agar for solid medium. Activated bacterial strains were selected with an inoculation loop and inoculated separately into the aforementioned media. *S. aureus*, *E. coli*, *B. subtilis*, and *S. typhimurium* cells were grown in LB at 37°C until the OD _600_ reached ∼0.4 and then diluted in six gradients using 10-fold serial dilution to prepare the test bacterial suspensions. Healthy, intact, and fresh leaves of *L. megaphylla* were sampled, sterilized with 75% ethanol for 10 s, washed three times with sterile water, dried, and sterilized under UV light for 20 min. Then, 0.1 mL of each prepared test bacterial suspension was inoculated onto the medium and evenly spread, and the Petri dishes were inverted. Then, 1 g of finely ground leaf powder was added to the lid of each Petri dish. The experiment was repeated three times. The control group did not contain leaf powder. *E. coli*, *S. typhimurium*, and *B. subtilis* were incubated in a 37°C incubator for 24 h, while *S. aureus* was incubated for 48 h. The bacterial growth on the plate medium was observed, colonies were counted, and the counts were compared with those of the blank control to determine the inhibition rate and assess the antibacterial effect.

The antifungal activity of the VOCs was evaluated with reference to previously described bacterial experimental methods. The antifungal efficacy was determined by calculating the inhibition rate based on the measured diameter of mycelial growth. Three types of fungi, *Rhizopus*, *Penicillium*, and *A. flavus*, were placed at the center of a 9 cm diameter Petri dish containing potato dextrose agar (PDA) medium in the dark at 30°C for 2–3 d to allow the mycelia to cover the substrate of the Petri dish. These processes were repeated three times. Subsequently, 7 mm diameter fungal disks were excised from each fungal culture and inoculated onto PDA medium. The Petri dishes were incubated at a constant temperature of 30°C for 3 d. The diameters of the nontreated and VOC-treated fungal disks were observed and measured.

### Construction and phenotypic determination of transgenic tobacco

The amplified *LmTPS1* coding sequence was inserted into the pCAMBIA2300-GFP vector, followed by enzyme digestion verification and PCR detection. The positive plasmids carrying the *LmTPS1* gene were subsequently transformed into *A. tumefaciens* GV3101 by electroporation [95]. Moreover, the *Agrobacterium*-mediated cotyledon transformation approach was applied to generate transgenic tomato lines overexpressing the *LmTPS1* gene, as described by Van *et al*. [97]. Kanamycin resistance and PCR were used to identify the transgenic tomato lines. After *in vitro* regeneration, all kanamycin-resistant plants were planted in soil and grown at 25°C with a 16/8 h light/dark photoperiod. Three to five mature leaves of 7-week-old T1-generation transgenic tomato lines (OE1, OE2, and OE3) were used to perform the pathogen inoculation experiments, following the method of Cui *et al* [98]. The *B. cinerea* strain was cultivated on potato dextrose agar (PDA) medium for one week until the hyphae fully covered the Petri dish. Then, an appropriate amount of hyphae were selected, dissolved in sterile distilled water, and thoroughly mixed to prepare for subsequent use. Meanwhile, the *Staphylococcus aureus* suspension was cultured until it reached an OD_600_ of 0.1. Next, 60 μl of each of the aforementioned bacterial suspensions was inoculated onto tomato leaves. Five days after inoculation, the phenotypes were observed and photographed using a super-depth-of-field microscope. The experiments were repeated three times, and all the results were similar. The β-caryophyllene standard, with a purity of ≥98%, was purchased from Shanghai Yuanye Bio-Technology Co., Ltd. The volatile compounds of the transgenic tomato lines and WT tomato plants in three tissues of *L. megaphylla* were detected using GC–MS/MS based on the previously described method for identifying volatile compounds but using a different sample-loading amount (0.5 g).

### Statistical analysis

Data analysis was conducted using SPSS software (v27.0). The significance of differences in volatile compound contents and gene expression levels across different samples were analyzed using one-way analysis of variance combined with a Tukey’s post hoc test (^*^P <0.05, ^**^P <0.01, ^***^P <0.001).

## Supplementary Material

Web_Material_uhaf116

## Data Availability

The raw sequences from the RNA-seq of *L. megaphylla* and the whole-genome sequence data reported in this paper have been deposited in the Genome Warehouse at the National Genomics Data Center (NGDC), Beijing Institute of Genomics, Chinese Academy of Sciences/China National Center for Bioinformation, with accession numbers CRA019464 and PRJCA031378, respectively.
