# Supplementary material for: High-quality Lindera megaphylla genome analysis provides insights into genome evolution and allows for the exploration of genes involved in terpenoid biosynthesis
Source: Hortic Res. 2025 Apr 29;12(8):uhaf116. doi: 10.1093/hr/uhaf116 (PMC12247515; doi:10.1093/hr/uhaf116)
Supplement: Web_Material_uhaf116 [file web_material_uhaf116.zip › Supplement Figures S1-8.docx]

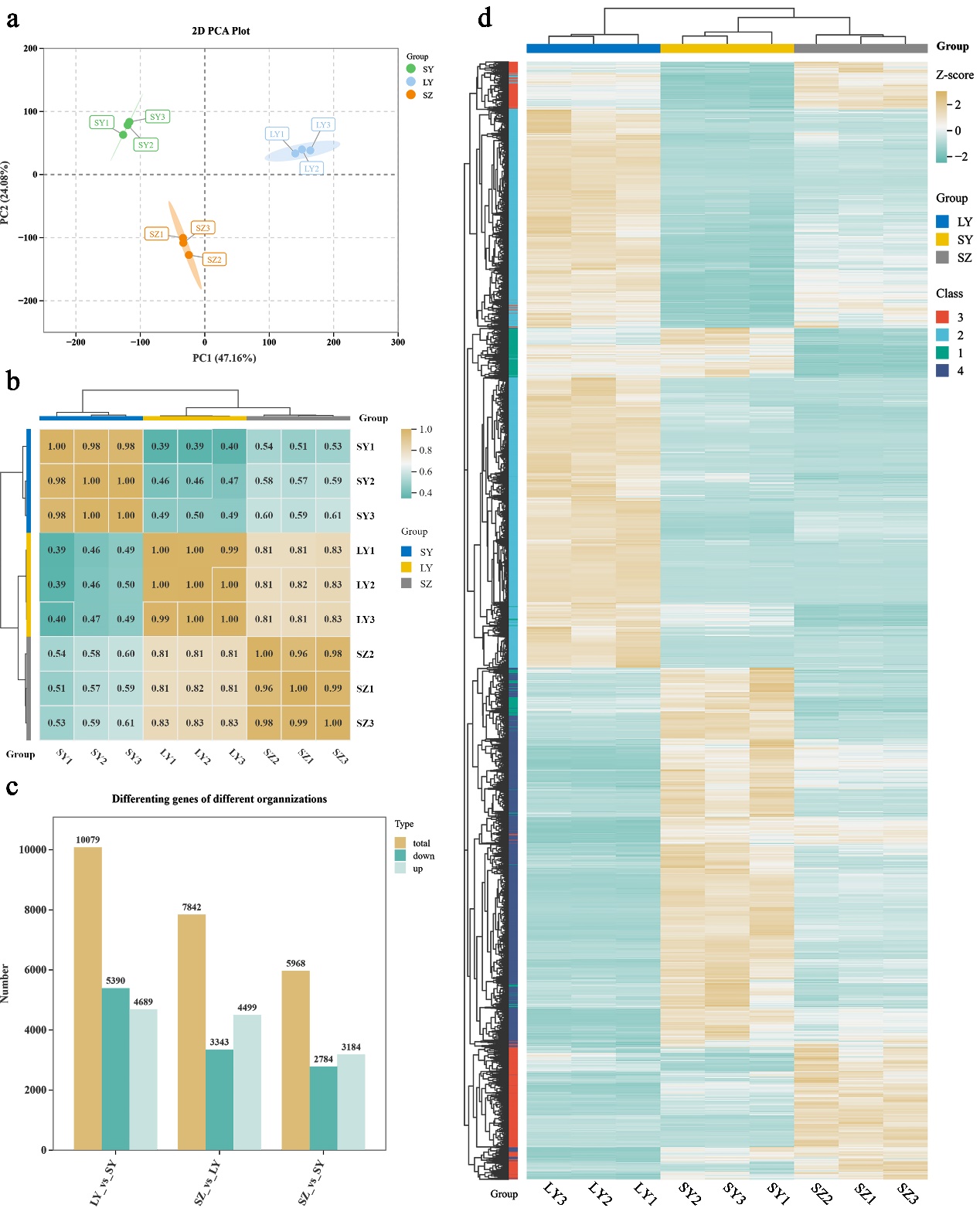


**Figure S1.** Transcriptome analysis of different tissues in *L.megaphylla*. (a) PCA of the transcriptome data. (b) Sample correlation analysis. (c) Number of DEGs under different tissues. (d) Clustering analysis of all DEGs across different tissue comparison groups.


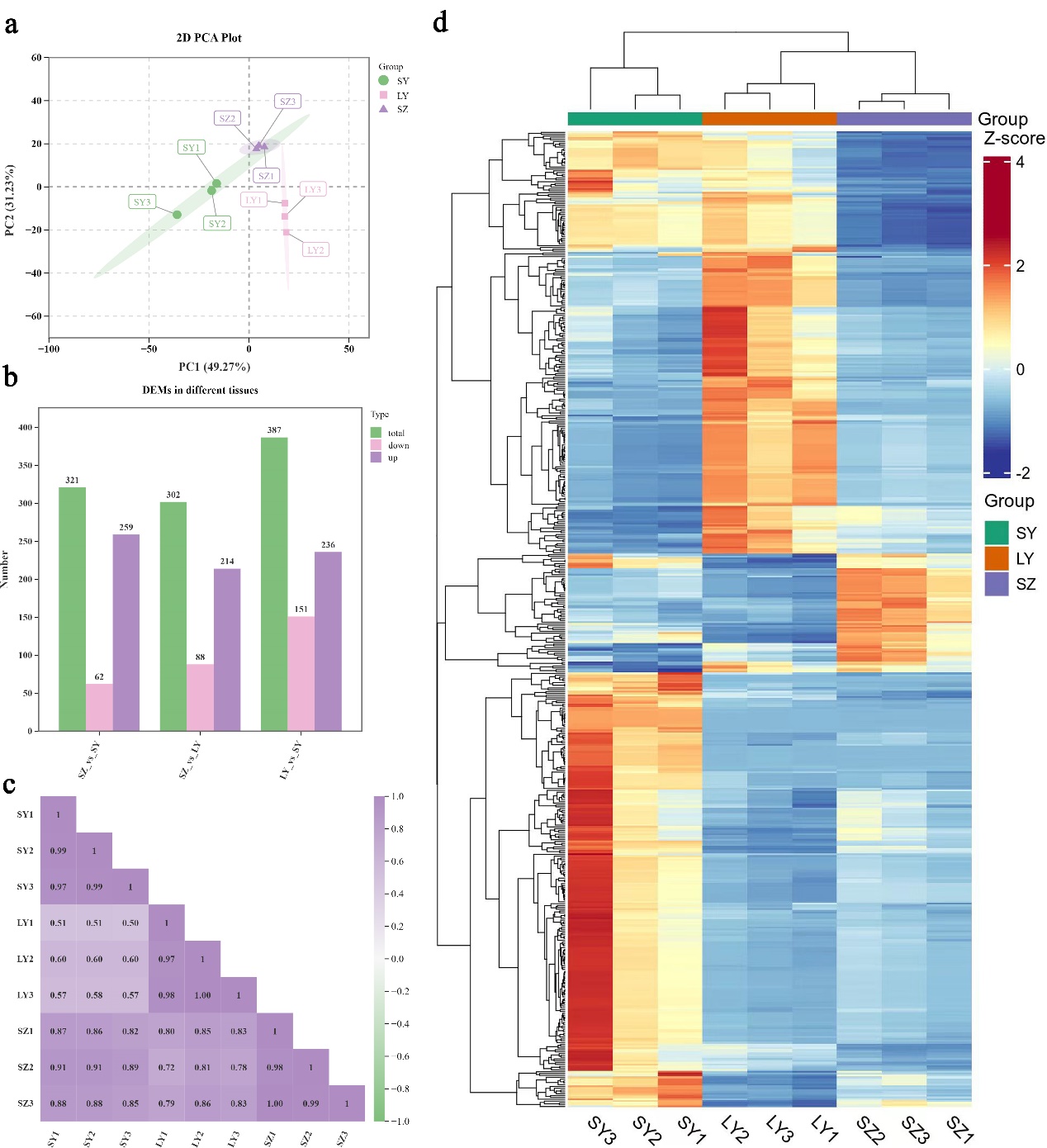


**Figure S2.** Metabolomic analysis of different tissues in *L.megaphylla*. (a) PCA of the Metabolome data. (b) Number of DAMs under different tissues. (c) Sample correlation analysis. (d) Clustering analysis of all DEM across different tissue comparison groups.


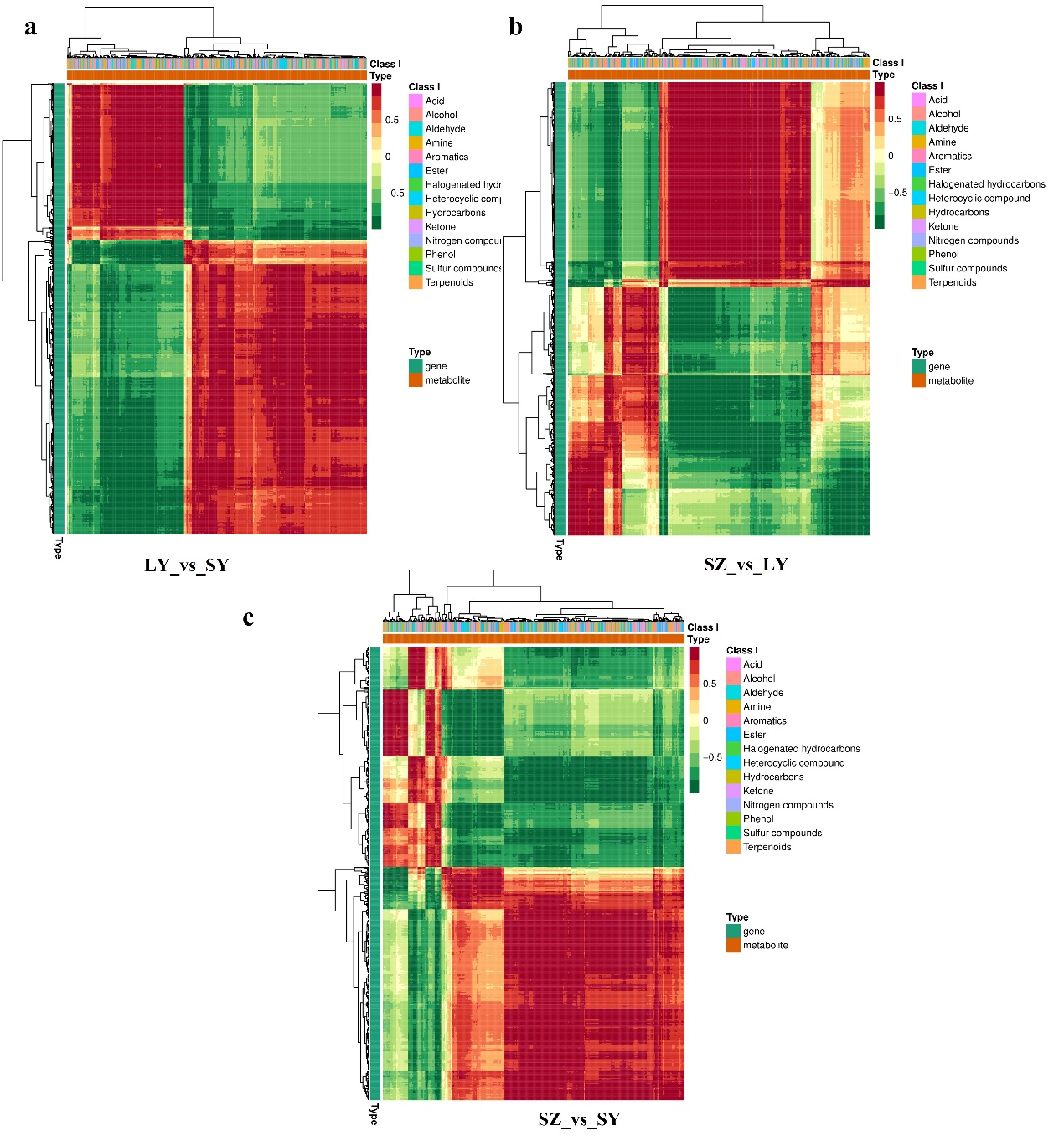


**Figure S3.** Heatmap analysis of the correlation between DEGs and DEMs in the comparisons of LY_vs_SY (a), SZ_vs_LY (b), and SZ_vs_SY (c).


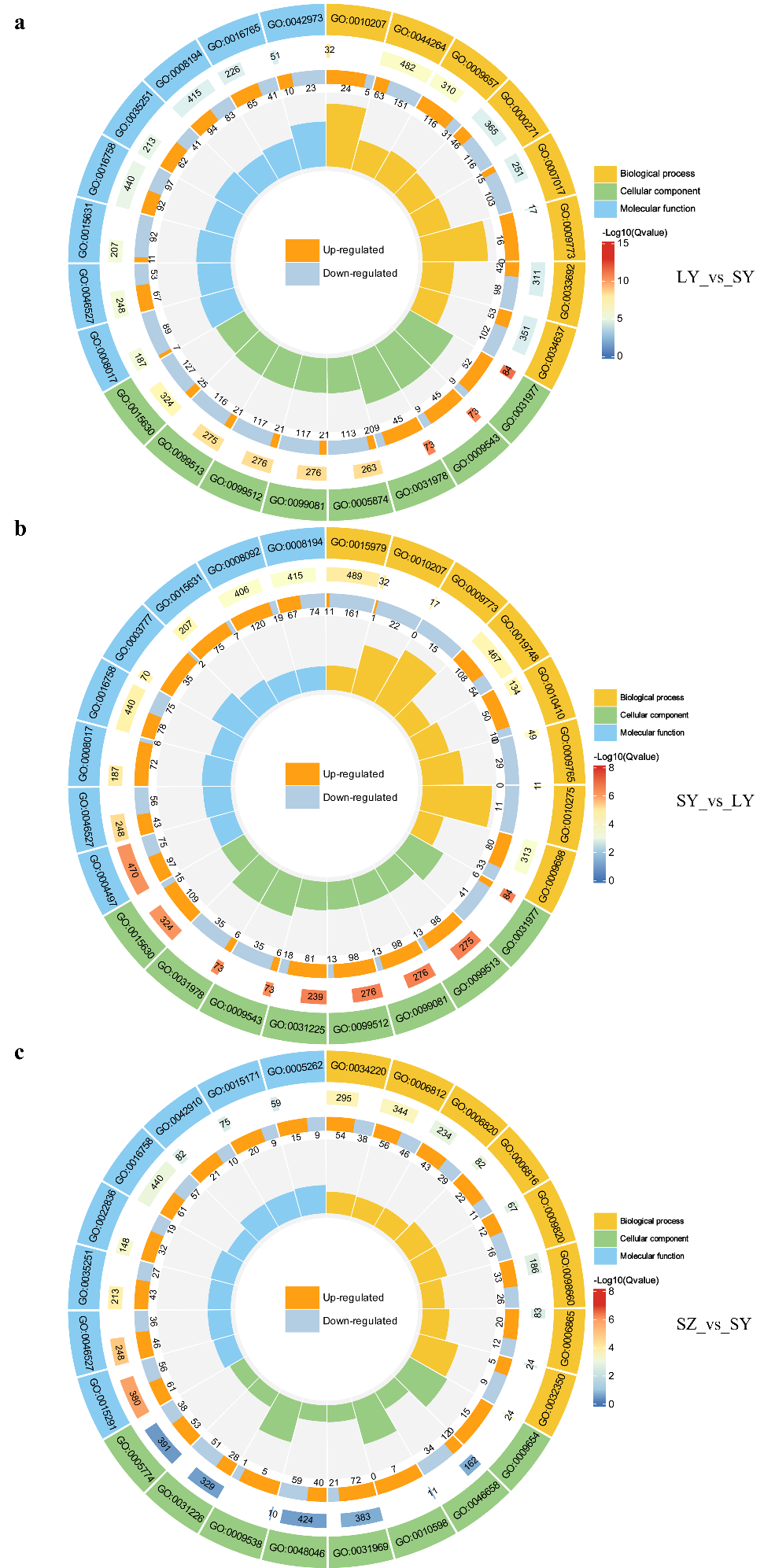
**Figure S4.** GO analysis of DEGs in the comparisons of LY vs. SY, SZ vs. LY, and SZ vs. SY in *L. megaphylla* (a-c).


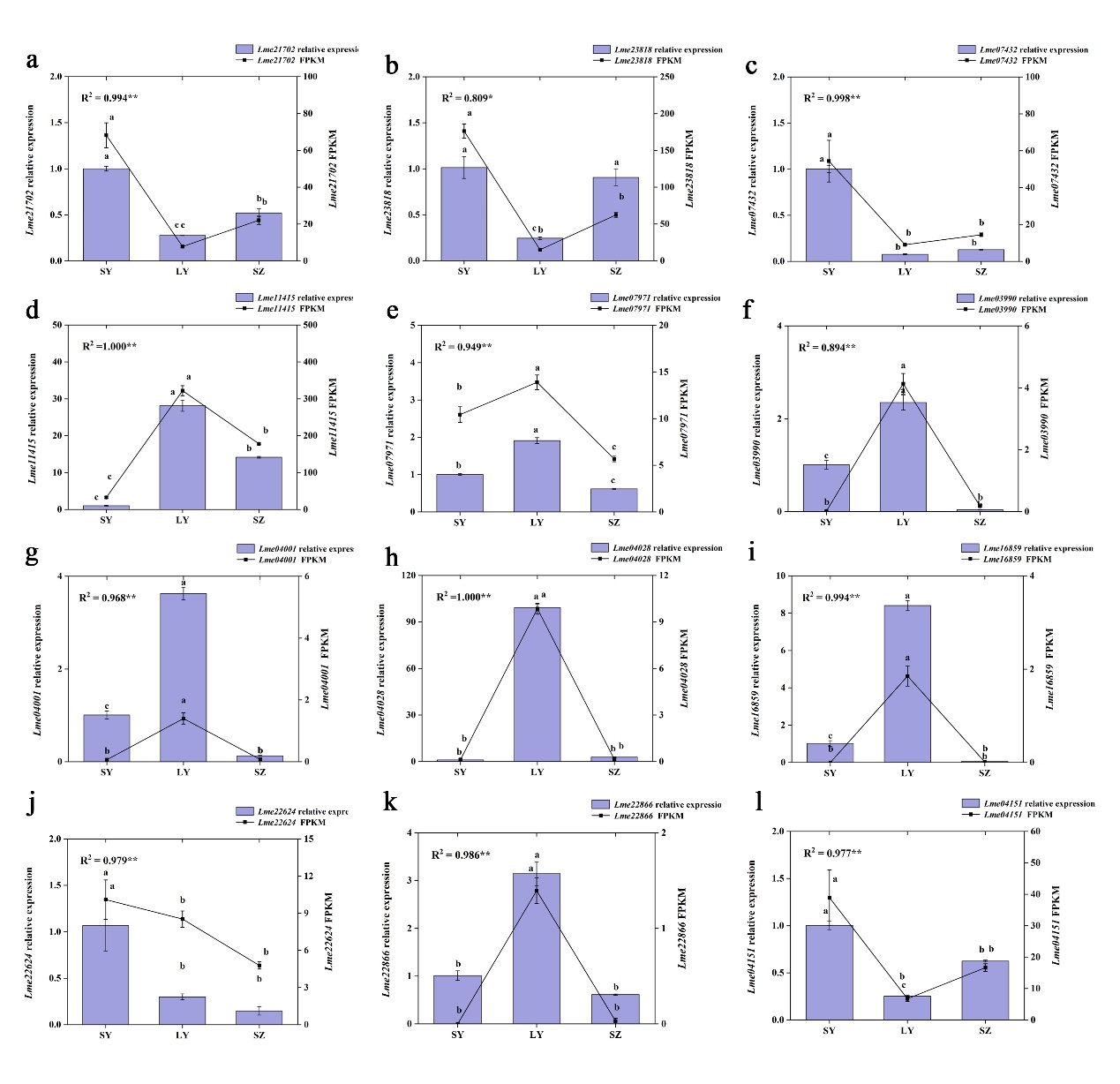
 **Figure S5.** Gene validation of RNA-seq with RT-qPCR. According to the LSD test, the average FPKM value and relative expression level are shown.

**
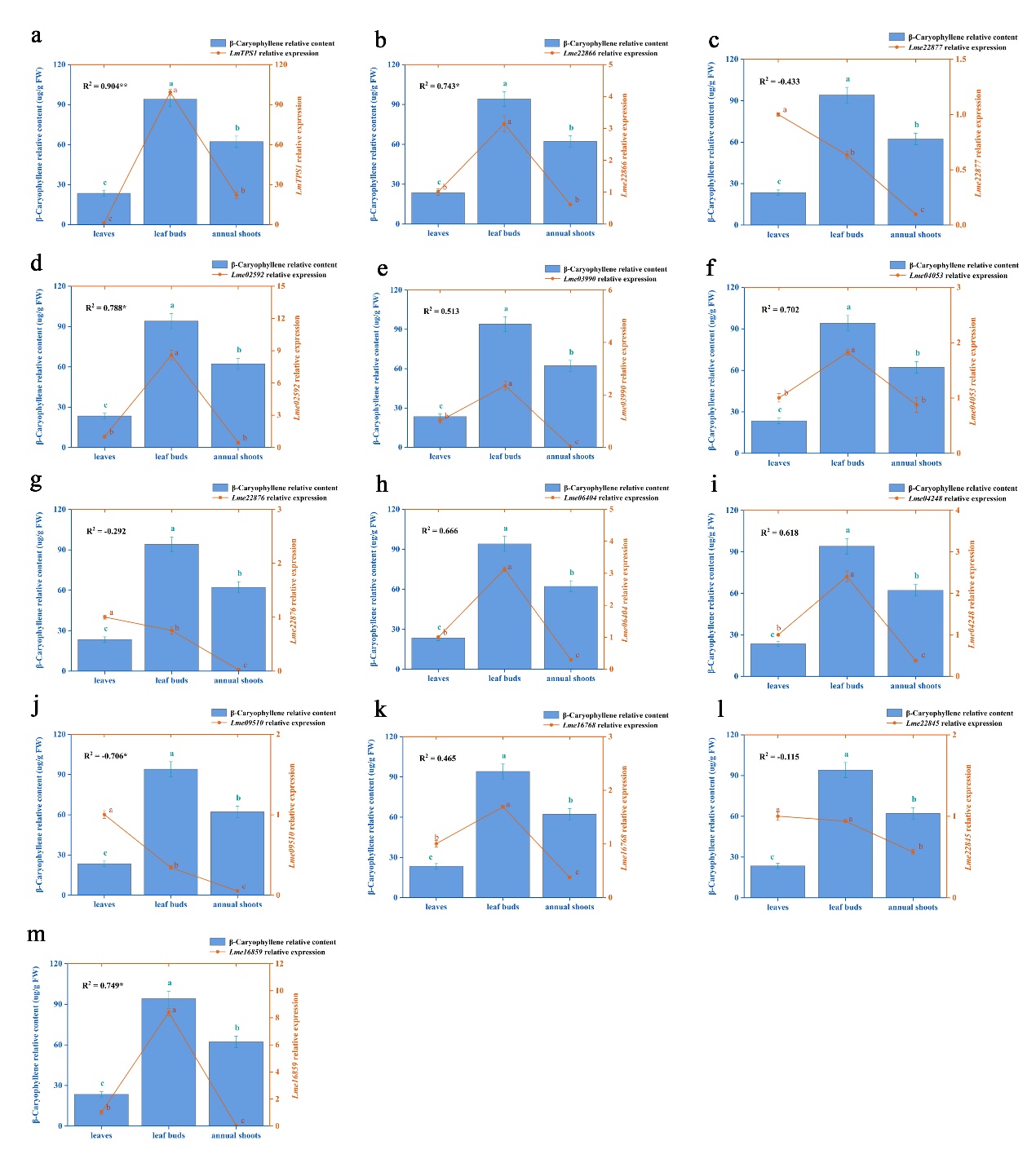
**

**Figure S6** Correlation analysis between 13 candidate genes and β-caryophyllene contents. (a-m) Column charts represent the β-caryophyllene contents in different organs; Line charts represent the expression level of 13 candidate genes. a, *LmTPS1*; b, *Lme22866*; c, *Lme22877*; d, *Lme02592*; e, *Lme03990*; f, *Lme04053*; g, *Lme22876*; h, *Lme06404*; i, *Lme04248*; j, *Lme09510*; k, *Lme16768*; l, *Lme22845* and m, *Lme16859*.


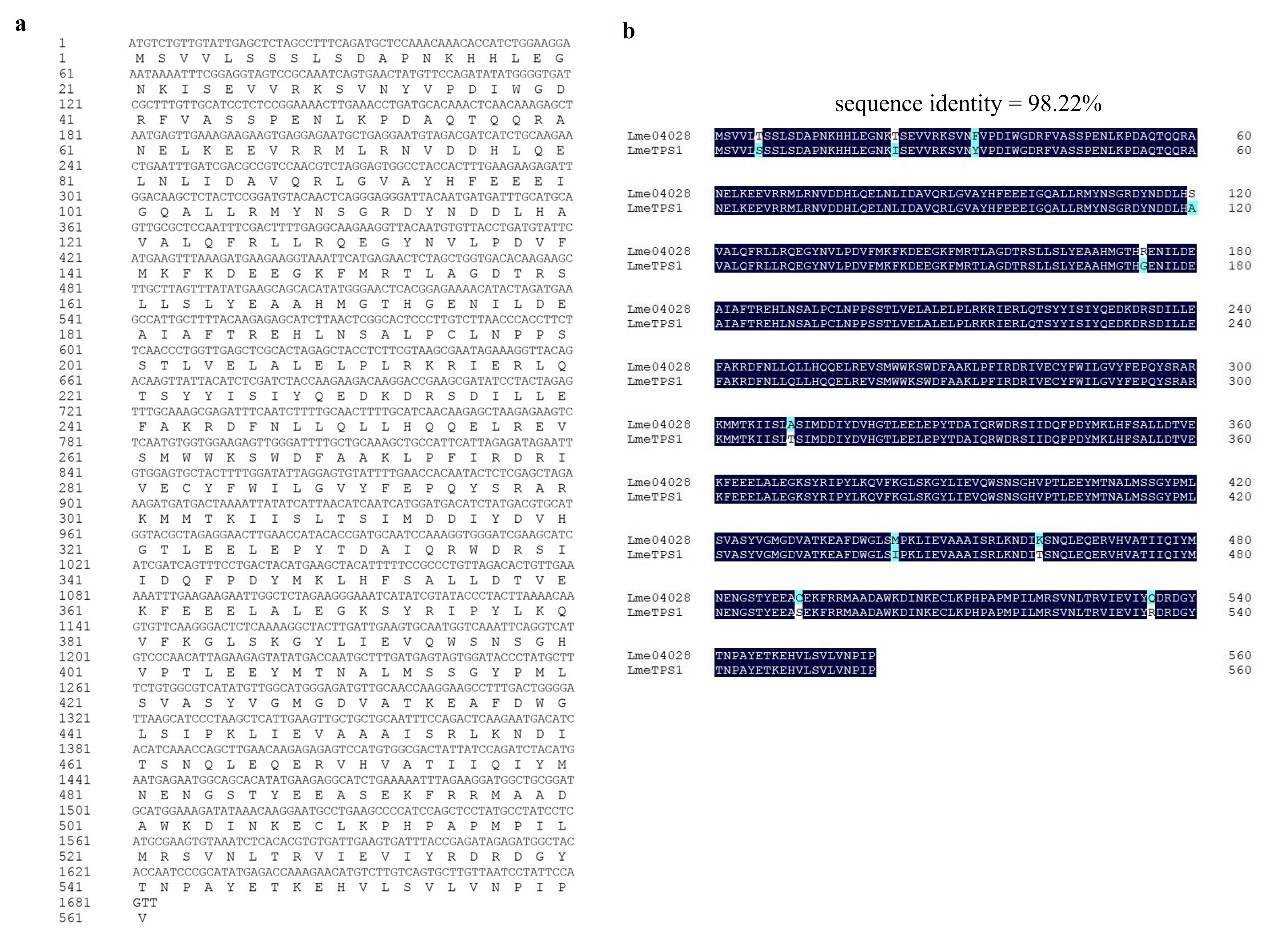


**Figure S7.** The full-length cDNA and deduced amino acid sequence of *LmTPS1* from *L. megaphylla* (a) and its homology comparison with the genomic amino acid sequence (b).


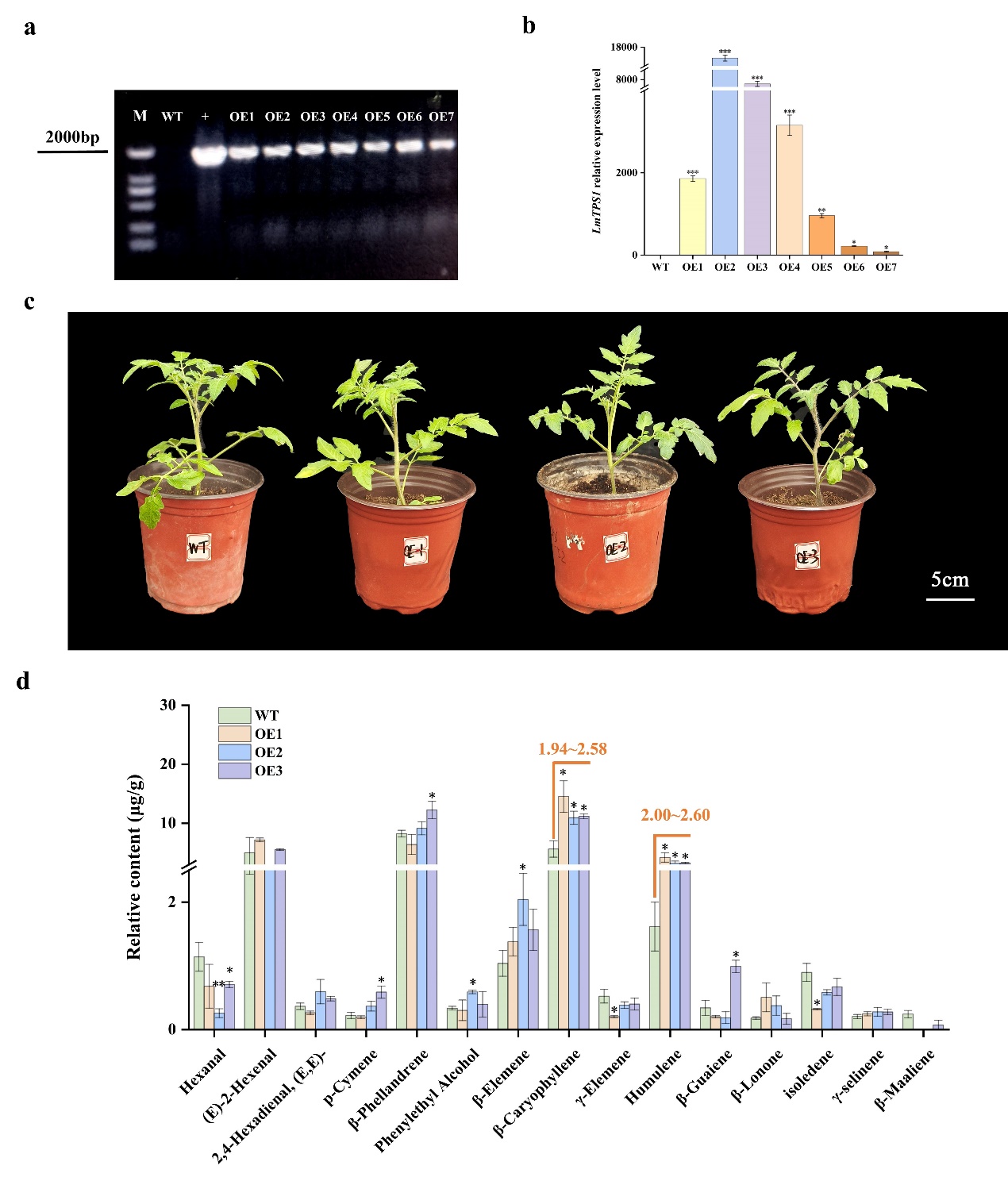


**Figure S8.** DNA detection of positive transgenic tomato seedlings (a) and RT-qPCR analysis of *LmTPS1* expression in positive transgenic tomato seedlings (b). The phenotype of WT tomato and LmTPS1-overexpressing transgenic tomatoes (OE#1, OE#2, and OE#3) (c). GC-MS measurement of endogenous volatiles in WT and transgenic tomatoes (OE#1, OE#2, and OE#3) (d).
